# Comparison between the rotary (Hyflex EDM^®^) and manual (k-file) technique for instrumentation of primary molars: a 12-month randomized clinical follow-up study*

**DOI:** 10.1590/1678-7757-2021-0527

**Published:** 2022-03-21

**Authors:** Andressa Cardoso Amorim, Amanda Valentim Caldeira, Samara Catarino Sampaio, Natalino Lourenço, Thais Marchini Oliveira, Denismar Alves Nogueira, Ana Beatriz da Silveira Moretti, Vivien Thiemy Sakai

**Affiliations:** 1 Universidade Federal de Alfenas Faculdade de Odontologia Departamento de Clínica e Cirurgia Alfenas Minas Gerais Brasil Universidade Federal de Alfenas (UNIFAL), Faculdade de Odontologia, Departamento de Clínica e Cirurgia, Alfenas, Minas Gerais, Brasil.; 2 Universidade de São Paulo Faculdade de Odontologia de Bauru Departamento de Odontopediatria, Ortodontia e Saúde Coletiva Bauru São Paulo Brasil Universidade de São Paulo (USP), Faculdade de Odontologia de Bauru, Departamento de Odontopediatria, Ortodontia e Saúde Coletiva, Bauru, São Paulo, Brasil.; 3 Universidade de São Paulo Hospital de Reabilitação de Anomalias Craniofaciais Bauru São Paulo Brasil Universidade de São Paulo (USP), Hospital de Reabilitação de Anomalias Craniofaciais, Bauru, São Paulo, Brasil.; 4 Universidade Federal de Alfenas Instituto de Ciências exatas Alfenas Minas Gerais Brasil Universidade Federal de Alfenas (UNIFAL), Instituto de Ciências exatas, Alfenas, Minas Gerais, Brasil.

**Keywords:** Dental pulp, Pulpectomy, Deciduous tooth

## Abstract

Rotational instrumentation is an alternative for the clinical practice of pediatric dentists. However, there are few records in the literature on the clinical and radiographic aspects of treated teeth over time.

**Objectives:**

Compare instrumentation time and filling quality between manual (k-file) and rotary (Hyflex EDM^®^) files, and clinically and radiographically follow-up the treated teeth for 12 months. Moreover, the characteristics of glass ionomer restorations and their interference in the treatment prognosis over time were evaluated.

**Methodology:**

In total, 40 children with pulp involvement in primary molars received treatment with Hyflex EDM^®^ or manual rotary files, performed by an operator. Clinical and radiographic aspects were observed at different times to determine the effectiveness of each technique.

**Results:**

The rotary system reduced instrumentation time when compared to the use of manual files (p≤0.05), but there was no difference in filling quality between the groups (p≥0.05). Moreover, both types of instrumentation were effective for 12 months (p≥0.05), and restoration retention influenced the emergence of periapical lesions (p≤0.05).

**Conclusion:**

Although rotary files reduce clinical time, the clinical and radiographic aspects of both techniques were similar over 12 months. Moreover, restoration retention has been shown to be related to treatment prognosis.

## Introduction

Despite advances in caries prevention measures and the reduction of its incidence rates worldwide, deep caries lesions that compromise pulp vitality remain a common occurrence in clinical practice.^1^ In these cases, pulp treatment (for example, pulpectomy) is essential to maintain the integrity of oral tissues, preserving deciduous teeth until their physiological exfoliation.^2^ However, the success of the pulpectomy procedure depends on an effective biomechanical preparation of the root canal system.^3^

This biomechanical preparation can be performed with rotary or manual files, and automated systems have been shown to significantly reduce instrumentation time, and more effectively clean and shape the root canal.^4,5,6^ However, other aspects contribute to the success of treatment. Some factors associated with clinical failure, such as the quality of root canal filling and coronal restoration, still need to be investigated.^7^

A recent systematic review of the clinical success of pulpectomy procedures in pediatric patients showed that there is no substantial evidence to determine whether instrumentation affects long-term clinical and radiographic success.^8^ Therefore, in this study, we compared instrumentation time and quality of root canal filling between manual and rotary techniques for the biomechanical preparation of primary molars, considering a follow-up period of 12 months. The characteristics of glass ionomer restorations and their interference with treatment prognosis were further examined. The null hypothesis of this study was that instrumentation with rotary files (Hyflex EDM^®^) would not be more effective than manual instrumentation (k-files) for treating pulpectomy in primary molars.

## Methodology

This study was previously approved by the Institutional Research Ethics Committee (protocol no. 3.071.573), registered on the Brazilian Clinical Trials Registry platform - ReBEC (registration no. RBR-5j25nm) and conducted following the Consolidated Standards of Reporting Trials (CONSORT). Legal guardians signed informed consent forms to authorize the participation of their children in the study. Sample size was determined based on previous data published in the literature.^9^ Considering a type I error (α) of 0.05 and a statistical power of 80%, a total sample size of 34 participants (n=17/group) was needed to detect any clinically significant difference of 5% between the groups. Thus, the final sample size consisted of 40 participants (n=20/group) to compensate for any sample loss during the follow-up period. The randomization method is further described in this section.

### Study design

This was a randomized clinical study whose experimental units were the primary teeth of children in need of endodontic treatment. The primary outcome was the instrumentation time required for root canal preparation (technique: one-way analysis). Secondary outcomes consisted of the quality of filling (technique: one-way analysis) and treatment success (technique and follow-up time: two-way analysis). The quality of coronal restorations was also determined over time. Of note, only the examiner was blind to the analysis, because there were remarkable differences in each technique which were impossible to mask to the operator or to study participants.

### Inclusion and exclusion criteria

Mandibular primary molars were selected based on the following inclusion criteria: presence of a deep carious lesion and pulp vitality, with pulp involvement on radiographic examination; provoked or spontaneous pain which was unresponsive to the use of analgesic drugs; provoked pain or absence of pain, with no hemostasis in a period of up to 5 min after pulpotomy and macroscopic signs of reversibility; absence of fistula or abscess and absence of bone rarefaction on radiographic examination, as well as absence of internal or external resorption of more than 2/3 of the root; and dental remnants which could be feasibly restored. Participants meeting the following criteria were excluded from the analysis: systemic diseases; teeth with less than 2/3 of the root remnant; teeth with mobility or rupture of the pericoronal follicle of the permanent successor; and cases in which restoration of the dental remnant was unfeasible. Only one tooth was eligible for each child.

### Randomization and allocation of study participants

Overall, 40 participants were allocated into two treatment groups, as follows: the experimental group (n=20), instrumented with Hyflex EDM^®^ rotary files, and the control group (n=20), instrumented with manual files. Patients were assigned sequential numbers during recruitment, and were allocated based on a previously set computer-generated randomized sequence. Treatment was completed in a single session and all procedures were performed by the same operator. Intra-examiner consistency and reliability were analyzed independently using the unweighted kappa test, with a score of 0.90 (excellent). All data were collected at the pediatric dentistry clinic of the Universidade Federal de Alfenas, between February 2019 and March 2020. Participants were enrolled by a member of the research team who also assigned the interventions. The random allocation sequence was generated by a second researcher, with the aid of a computer.

### Intervention protocol

Pulpectomy procedures were performed under local anesthesia of the mandibular alveolar nerve and rubber dam isolation. The carious tissue was removed with a dentin spoon, followed by cavity opening with 1014-1015 spherical diamond tip drills (Kg Sorensen, Barueri, São Paulo, Brazil) in high rotation under irrigation. The root canal was explored using a K-file #10 (Maillefer Instruments, Ballaigues, Switzerland) and working length was determined by passively inserting the file in each root canal with a rubber stopper. When the tip of the file was at the apical foramen height, the rubber stopper was leveled with the respective cusp tip and the length of each root canal was recorded. Working length was obtained by subtracting two millimeters from the total length of the root canal. Thus, the primary outcome of this study was the time used to instrument root canals, and its secondary outcomes were the analysis of the quality of filling and restoration, as well as their clinical and radiographic aspects during follow-ups. To assess the primary outcome, a stopwatch was used, and clinical and radiographic examinations were performed to analyze secondary outcomes. Only the evaluator was susceptible to blinding, since he was the only one who had no contact with the patients or participated in the procedures.

### Root canal preparation

In the control group, biomechanical preparation of the root canal was performed by the conventional method (manual technique) with stainless steel K-files #15 to #30 (Dentsply Maillefer, OK, USA), using the quarter-turn-and-pull technique. In the experimental group, Hyflex EDM^®^ files (Coltene / Whaledent, Allstätten, Switzerland) were used for rotary instrumentation using 25/.12, 10/.05, and 25/~ taper files. Instrumentation started with the 25/.12 file to shape the cervical third; then, the 10/.05 file (Glidepath) was used for an initial exploration of the apical third; lastly, the 25/~ file variable taper was used to complete the preparation of the apical third. Rotary files were used on an X-Smart engine (Dentsply-Maillefer, OK, USA) operating at 500 rpm with a torque of up to 2.5 Ncm (25 mnm), except for the Glidepath files, which were used at 300 rpm with a torque of up to 1.8 Ncm (18 mnm). In both groups, canals were cleaned and shaped by the “crown-down” technique using progressively larger conical files. In between each instrument change, canals were irrigated with 1 ml of 1% NaOCl. Therefore, at each instrument change, 3 ml of NaCL solution were used. Irrigations were performed with a 30-G needle placed 2 mm before the working length. Thus, at the end, 12 ml of 1% NaOCl were used in each tooth in both groups. All irrigation procedures were performed with a 30-G needle placed 2 mm before the working length. Each instrument was replaced according to the manufacturer’s recommendations. The time spent during biomechanical preparation was recorded on a clinical chart.

### Root canal filling

**Figure 1:**
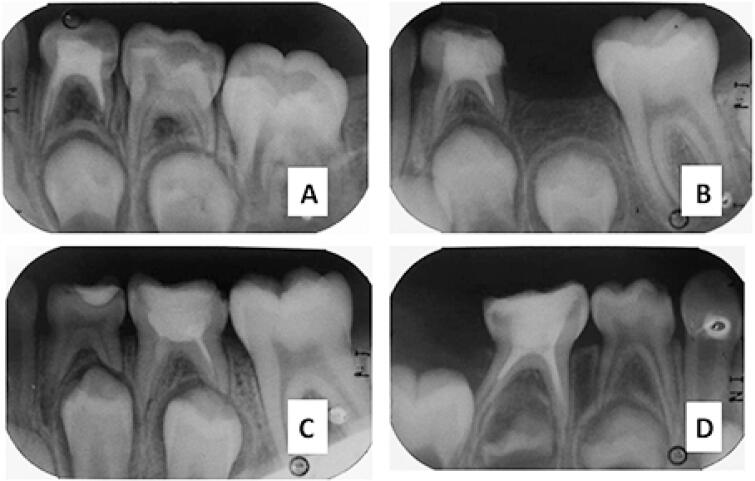
A) Satisfactory filling in tooth 74, instrumented with a manual file. B) Satisfactory filling in tooth 74, instrumented with a rotary file. C) Unsatisfactory filling in tooth 85, instrumented with a manual file. D) Unsatisfactory filling in tooth 75, instrumented with a file roundabout

After final irrigation with a saline solution, root canals were dried with paper tips and filled with a mixed paste composed of calcium hydroxide and polyethylene glycol (Calen^®^ - SS White, São Paulo, SP, Brazil) thickened with zinc oxide (slow curing - Biodinâmica Quím. e Farm. Ltda, Ibiporã, PR, Brazil). A pressure syringe and a manual file were used to push the paste into the apex. The quality of the root canal filling was classified as satisfactory, underfilled or overfilled. Roots in which the filling paste reached either the instrumentation limit or the root apex were considered “acceptable”; roots in which the material was placed before the instrumentation limit were considered “insufficiently filled”; and cases with material leakage to the periapex were considered “overfilled”(Figure 1). Analysis of the quality of root canal filling was performed separately for mesial and distal roots.

### Coronal restoration

Excess filling material was removed and the coronal space was covered with a Coltosol^®^ liner (Vigodent, Rio de Janeiro, Rio de Janeiro, Brazil) and restored with resin-modified glass-ionomer cement (Vitremer^®^, 3M ESPE Produtos Dentários, Sumaré, SP, Brazil), which was light-cured for 40 s. Coronal fillings were examined during clinical follow-up with the aid of an exploratory probe and a clinical mirror under cotton roll isolation and reflector lighting, according to the modified criteria of the United States Public Health Service (USPHS).^10,11^

### Participant follow-up

Patients were followed up after three, six, and 12 months after the pulpectomy procedure to assess the presence of pain, fistula or abscess, pathological mobility, and sensitivity to percussion. A periapical radiograph was obtained to assess the presence of a radiolucent inter-radicular area, the periodontal ligament condition, and the presence of periapical lesion(s). Radiographic assessment was considered successful when teeth showed no radiolucency in the inter-radicular area, no periapical lesion, an intact periodontal ligament, and satisfactory root canal filling. Assessment of coronal restorations followed the USPHS criteria.

### Statistical analysis

Quantitative data were analyzed by the Mann-Whitney test and qualitative data were analyzed by the chi-squared test. Generalized estimation equations (GEE) were used to check for differences in the longitudinal data. A 5% significance level was considered (a≤0.05) in two-tailed tests. All statistical tests were carried out in SPSS version 20.0 (Armonk, NY, United States).

## Results

**Figure 2:**
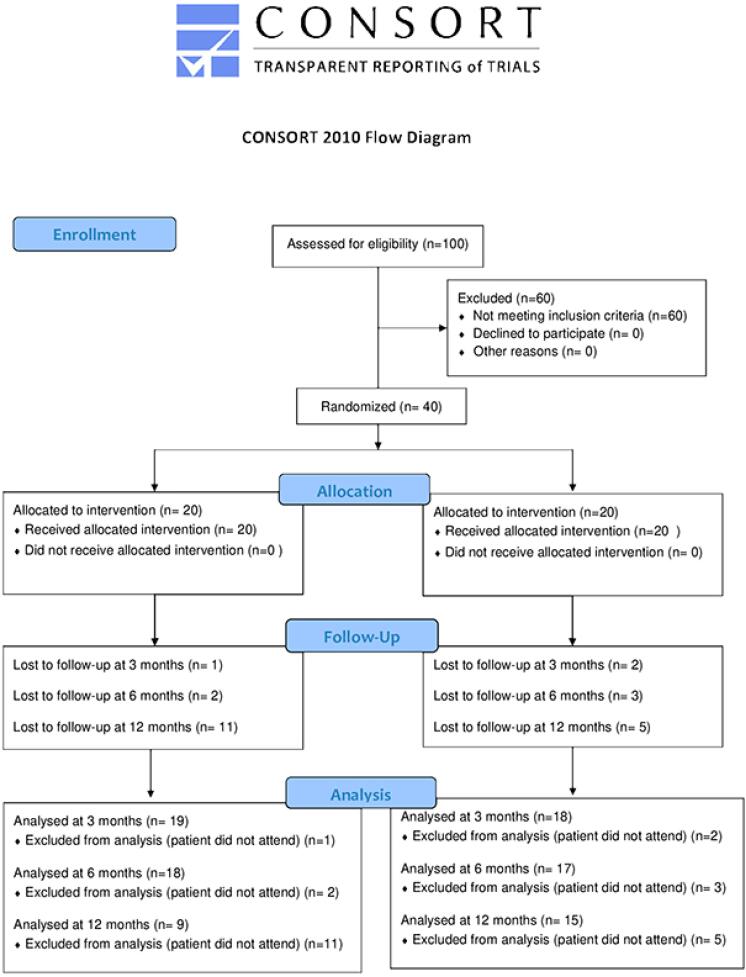
Flow diagram of the study according to the Consolidated Standards of Reporting Trials (CONSORT) guidelines (2010)

Table 1 describes the demographic characteristics of the sample. CONSORT guidelines were followed for planning and reporting study outcomes, as shown in Figure 2. There were no differences between groups regarding age and sex. As shown in Table 2, the mean instrumentation time in the control group (manual technique) was significantly longer than in the experimental group (rotary system) (Mann-Whitney test, p≤0.05). However, no significant difference in the quality of root canal filling was observed between the groups (Chi-square test, p≥0.05, Table 3).

**Table 1:** Demographic characteristics of the patients and samples included in the study

Variable	f	%
**Age Group**		
04 to 07 years	17	42.5%
08 to 11 years	23	57.5%
Gender		
Female	12	30.0%
Male	28	70.0%
**Tooth selected**		
First Left Molar (74)	14	35.0%
Second Left Molar (75)	8	20.0%
First Right Molar (84)	9	22.5%
Second Right Molar (85)	9	22.5%

**Table 2:** Analysis of the instrumentation time required for the biomechanical preparation of root canals and standard deviation by manual and rotary techniques

Groups	Time (minutes)	Standard deviation	p-value
Manual	20.24	± 5.157	0.001*
Rotary	11.30	± 3.230	

(*) Significant at 5% by the Mann–Whitney test

**Table 3:** Analysis of the quality of root canal filling, according to the instrumentation technique

Satisfactory Fillings			
	Mesial root	Distal root	p-value
Manual	13 (65.0%)	14 (70.0%)	0.125
Rotary	16 (80.0%)	15 (75.0%)	

Both groups were treated successfully and no postoperative pain, sensitivity to percussion, abscess/fistula, secondary caries or pathological mobility were observed during the 12-month follow-up in any of the groups. As shown in Table 4, there was no significant difference in radiographic success and failure rates between teeth treated with manual and rotary files (GEE analysis, p≥0.05). The variables indicating radiographic failure can be found in Table 5. Overall, there was no significant difference between radiographic failure rates in both groups over 12 months (GEE analysis, p≥0.05, Table 5).

**Table 4:** Radiographic success and failure rates over a 12-month follow-up of primary teeth endodontically treated with manual and rotary systems

	Event rates for each assessment					
	3 months		6 months		12 months	
	Success	Failure	Success	Failure	Success	Failure
Manual	17 (94.4%)	1 (5.6%)	15 ( 88.2%)	2 (11.8%)	14 (93.3%)	1 (6.7%)
Rotary	16 (84.2%)	3 (15.8%)	14 (77.8%)	4 (22.2%)	6 (66.7%)	3 (33.3%)

**Table 5:** Radiographic findings of primary teeth endodontically treated with manual and rotary systems over a 12-month follow-up

	3 months		6 months		12 months		
	Manual	Rotary	Manual	Rotary	Manual	Rotary	p-value
Periapical injury	1(25.0%)	3 (30.0%)	2 (50.0%)	4 (40.0%)	1 (25.0%)	3 (30.0%)	0.080
Radiolucent area	1(25.0%)	3 (30.0%)	2 (50.0%)	4 (40.0%)	1 (25.0%)	3 (30.0%)	0.080
Periodontal ligament without integrity	1(25.0%)	3 (30.0%)	2 (50.0%)	4 (40.0%)	1 (25.0%)	3 (30.0%)	0.080

The data shown in Table 6 revealed no significant differences between the groups concerning the integrity, retention, discoloration, anatomical shape, and roughness of the coronal restorations over time (GEE analysis, p≥0.05 for all variables). However, restoration retention was significantly correlated with the occurrence of periapical lesions, regardless of the instrumentation technique (p≤0.05, Table 7).

**Table 6:** Characteristics of coronal restorations over time in primary teeth endodontically treated with manual and rotary systems

Characteristics	3 months	6 months	12 months
**Integrity**			
Alpha	32 (39.5%)	27 (33.3%)	22 (27.2%)
Bravo	3 (27.3%)	6 ( 54.5%)	2 (18.2%)
Charlie	2 (33.3%)	3 ( 50.0%)	1 (16.7%)
**Retention**			
Alpha	33 (40.2%)	27(32.9%)	22 (26.8%)
Bravo	2 (20.0%)	6 (60.0%)	2 (20.0%)
Charlie	2 (33.3%)	3 (50.0%)	1 (16.7%)
**Discoloration**			
Alpha	35 (38.0%)	33 (35.9%)	24 (26.1%)
Bravo	1 (50.0%)	1 (50.0%)	0 (0.0%)
Charlie	1 (25.0%)	2 (50.0%)	1 (25.0%)
**Anatomical shape**			
Alpha	36 (38.3%)	34 (36.2%)	24 (25.5%)
Bravo	0 (0.0%)	0 (0.0%)	0 (0.0%)
Charlie	1 (25.0%)	2 (50.0%)	1 (25.0%)
**Roughness**			
Alpha	35 (40.2%)	28(32.2%)	24 (27.6%)
Bravo	1 (14.3%)	6 (85.7%)	0 (0.0%)

**Table 7:** Correlational analysis between restoration retention and the occurrence of periapical lesion

Retention	Periapical injury	p-value
**3 months**		
Alpha	2 (6.1%)	0.028*
Bravo	1 (50.0%)	
Charlie	1 (50.0%)	
**6 months**		
Alpha	2 (7.4%)	
Bravo	4 (80.0%)	0.001*
Charlie	0 (0.0%)	
**12 months**		
Alpha	2 (9.5%)	
Bravo	1 (50.0%)	0.025*
Charlie	1 (100.0%)	

(*) Significant at 5% by the Chi-Square test

## Discussion

Technological advances have contributed to the development of new endodontic instrumentation techniques.^10,12^ For instance, in the year 2000, nickel-titanium (NiTi) automated files were incorporated into pediatric dental care,^11^ with the advantage of having better predictability, shorter instrumentation time, and generating less in-office stress for the patient.^13^

Consistent with the scientific literature, our study indicates that the use of rotary instrumentation (versus the manual technique) may optimize clinical performance by reducing instrumentation times, which is a relevant aspect to be considered for pediatric patients. As biopulpectomy procedures are complex, reducing chair time prevents the fatigue of both the patient and the dentist.^14^ Although several studies agree that automated instrumentation reduces the instrumentation time for root canal preparation,^13,15,16,17,18^ not all evidence is from randomized clinical trials and some results were obtained only in permanent teeth. Thus, even though principles and approaches are similar for both primary and permanent dentitions, some aspects, such as tooth anatomy, and patient acceptance and cooperation, among others, must be considered before generalizing the results. Therefore, instrumentation time in each study may be different depending on the operator’s skill and the patient’s behavior. Additionally, rotary files are limited to a smaller series with greater cutting potential, which may altogether reduce working time.

The removal of infective bacteria and their substrates by instrumentation alone is limited. The anatomical complexities of root canals and the limited access of therapeutic agents to the microchannel system are challenging. Therefore, the irrigation solution used during instrumentation helps to clear debris from the instrumented canals, dissolving organic tissue residues, disinfecting the root canal space, reducing oxidization during instrumentation, and removing the smear layer without irritating biological tissues.^19^ Furthermore, the effectiveness of irrigation solutions is directly related to their concentration and volume.^20^ In this study, the main irrigating antibacterial solution was 1% sodium hypochlorite (NaOCl), due to its antimicrobial efficacy consolidated in the literature.^21,22,23^ The use of NaOCl contributed to success rates in both types of instrumentation.

Moreover, these instruments must properly model the root canal to facilitate the introduction of filling material.^25^ Periapical radiographs of treated canals submitted to rotary instrumentation commonly display a more regular and conical shape, as previously described in the literature.^16^ The conical shape produced by rotary files facilitates the filling of the root canal system.^6,24,25^ Yet, in our study, despite the examiner’s subjective perception, both instrumentation techniques were effective in terms of root canal filling. Our results agree with those of Kummer, et al.^26^ (2008) and Azar, et al.^15^ (2012), who showed a similar performance in the cleaning capacity and quality of filling of root canals submitted to manual and rotary instrumentation. In contrast, other studies have found that rotary systems provide better quality of filling.^16,17,23^ Quality of filling is not the only parameter determining the effectiveness of endodontic treatment; other clinical signs of endodontic success include absence of pain and swelling, absence of drainage and fistula, functional teeth with normal periapical physiology, and absence or remission of periapical bone rarefaction^27^. Therefore, clinical and radiographic follow-up examination is utterly important. Post-treatment failures in the quality of filling and coronal restoration may be a risk factor leading to residual infection.^28^ Regrettably, there is insufficient evidence published in the literature addressing the follow-up of children submitted to the same instrumentation techniques tested in this study, which makes it difficult to compare findings.

In our study, there was no report of postoperative pain, sensitivity to percussion, abscess/fistula, secondary caries or pathological mobility, indicating that the treatment of participants was successful in both groups. However, symptom-based diagnosis in children is imprecise and limited^29^ since they are unable to accurately provide subjective information, such as pain.^30^ In contrast with our findings, Morankar, et al.^31^ (2018) reported higher clinical success rates in the group treated with manual files at six and 24 months (at these time points, clinical success rates were 85.2% and 92.3% for the rotary and manual techniques, respectively). However, a study by Elheeny, Khattab, and Fouda^32^ (2015) reported that the rotary system outperformed the manual technique, with higher clinical success rates at six months.

Our findings indicated that, although the group treated with rotary files showed a higher frequency of periapical lesions, radiolucent inter-radicular areas, and disruptions of periodontal ligaments, there was no significant difference between the instrumentation techniques. Morankar, et al.^31^ (2018) showed that the manual technique performed slightly better at the 6-month follow-up. However, at the 24-month follow-up, radiographic success rates were 66.7% for the rotary system and 65.4% for the manual technique. Only three clinical trials longitudinally evaluated the success of rotary and manual techniques for root canal treatment in primary teeth^27,31,32^. Regrettably, one of these studies^9^ failed to report the success of different techniques and only showed the results for instrumentation times. Therefore, this study was assigned a high risk of bias due to the selection of reported results. Despite the limited number of trials, rotary and manual techniques showed similar success rates in the endodontic treatment of primary teeth, with a moderate level of evidence due to imprecision^33^. In our study, the higher – albeit non-significant - frequency of periapical lesions in the experimental group must be interpreted considering its clinical implications. The radiographic failures observed in the experimental group may be related to the accumulation or overflow of dentin shavings in the periapical region, which was further exacerbated by the rhizolysis of primary teeth. Liu, et al.^34^ (2013) reported that, despite the various clinical advantages of rotary and reciprocating instrumentation systems over the manual technique, the former may increase stress within the root canal. Furthermore, other factors may also be associated with the occurrence of periapical lesions, such as contamination during instrumentation, selection of sealing material, and quality of the final restoration.

Based on the USPHS criteria, the characteristics of resin-modified glass ionomer restorations were satisfactory in most treated teeth. Some patients with more difficult management, or who had a subgingival class II cavity, needed repair or an additional restoration – in cases in which failures were clinically detected during follow-up (Charlie criterion). Monitoring final restorations is of great importance for the prognosis of endodontic treatment. In our study, there was a statistically significant relation between restoration retention and the onset of periapical lesions over time. The hermetic sealing of the cavity prevents microleakage and bacterial contamination inside the root canals. Therefore, a good alternative for these specific cases, which was previously proposed by Garg, et al.^35^ (2016), is the use of precast metal crowns, which (i) protect the residual tooth, possibly damaged after excessive caries removal; (ii) are cost-effective in the long term; and (iii) have a low failure rate due to their hermetic sealing. In this study, the difference in the number of cases with radiographic success was insignificant between the groups at three, six, and 12 months, thus showing that both instrumentation techniques were effective. Moreover, there was no harm or unwanted effect during this study.

The main limitation of this study lies in its small sample size, which failed to allow the detection of small differences in qualitative variables. This study was designed with a focus on its primary outcome, so the results of secondary outcomes should be interpreted with caution, although they are of great clinical relevance. The analysis of secondary results is prone to type II errors due to lack of statistical power.^36^ A major difficulty of our study consisted of recruiting eligible children, which resulted in a small sample size and a relatively wide age group. Importantly, there was a considerable sample loss during the last follow-up due to the Covid-19 pandemic, which interrupted this study. Another limitation to be considered was the impossibility of blinding the operator and participating children due to the remarkable differences in instrumentation techniques.

The use of rotary files in pediatric dentistry warrants further investigation. Although several studies have already showed the possibility of using different file brands and series, other aspects of automated systems are yet insufficiently explored, such as their cleaning efficacy (microbial reduction), children’s behavior during treatment, operator’s satisfaction, file resistance and flexibility, among others. Thus, further longitudinal clinical studies with larger sample sizes are needed to establish guidelines or clinical protocols for the safe use of automated instrumentation systems in pediatric dental care.

## Conclusion

Within the limitations of this study, it is concluded that both techniques showed good clinical and radiographic results during the 12-month follow-up, but rotary files allowed a faster instrumentation of the root canal system in primary molars. Furthermore, our findings indicated that failures in coronal restorations were related to the emergence of periapical lesions during the follow-up period.
